# *Clostridium botulinum* and *Clostridium perfringens* Occurrence in Kazakh Honey Samples

**DOI:** 10.3390/toxins11080472

**Published:** 2019-08-13

**Authors:** Balgabay Maikanov, Raikhan Mustafina, Laura Auteleyeva, Jan Wiśniewski, Krzysztof Anusz, Tomasz Grenda, Krzysztof Kwiatek, Magdalena Goldsztejn, Magdalena Grabczak

**Affiliations:** 1 Department of Veterinary Sanitation, Faculty of Veterinary Medicine and Technology of Animal Husbandry, S. Seifullin Kazakh Agro Technical University, Zhenis Avenue 62, 010011 Astana, Republic of Kazakhstan; 2 Departament of Food Hygiene and Public Health Protection, Faculty of Veterinary Medicine, Warsaw University of Life Sciences—SGGW (WULS-SGGW), ul.Nowoursynowska 159, 02-776 Warsaw, Poland; 3 Department of Hygiene of Animal Feeding Stuffs, National Veterinary Research Institute, Partyzantow 57, 24-100 Pulawy, Poland

**Keywords:** *C. botulinum*, *C. perfringens*, honey, Kazakhstan

## Abstract

The aim of this study was to assess occurrence of *Clostridium botulinum* and *Clostridium perfringens* in honey samples from Kazakhstan. Analyses were carried out using a set of PCR methods for identification of anaerobic bacteria, and detection of toxin genes of *C. botulinum* and *C. perfringens*. Among 197 samples, *C. botulinum* was noticed in only one (0.5%). The isolated strain of this pathogen showed the presence of the *bont/A* and *ntnh* genes. *C. perfringens* strains were isolated from 18 (9%) samples, and mPCR (multiplex PCR) analysis led to them all being classified as toxin type A with the ability to produce α toxin. Sequence analysis of 16S rDNA genes showed occurrence in 4 samples of other anaerobes related to *C. botulinum*, which were *C. sporogenes* and *C. beijerinckii* strains. *C. botulinum* prevalence in honey samples from Kazakhstan in comparison to the prevalence in samples collected from the other regions seems to be less. The highest prevalence of *Clostridium* sp. was noticed in the East Kazakhstan province. Our study is the first survey on BoNT-producing clostridia and *C. perfringens* prevalence in Kazakh honey.

## 1. Introduction

Honey is a natural sweet substance produced by bees from nectar, blossoms or from the secretion of parts of plants or their excretions. Primarily, honey consists of sugar and water, with sugar making up 95–99% of the dry matter. The majority of sugars are monosaccharides and they constitute 85–95% of total sugars. Of these, fructose (38.2%) and glucose (31.3%) are the major components [1,2,3].

Microorganisms present in honey are those which are able to survive in a high concentration of sugars and acidity and where antimicrobial substances occur. The primary sources of microbial contamination are likely to include pollen, the digestive tracts of honeybees, dirt, dust, air, and flowers. Secondary sources of microbial contamination in honey are humans, equipment, containers, wind, dust, etc. [1,4,5].

A high concentration of sugars and the presence of antimicrobial substances are not favorable conditions for survival and growth of vegetative microflora. However, even in such environments the presence of anaerobic spore-forming bacteria is possible. Among the durable bacteria are pathogenic Clostridia which pose a risk of human infection or toxicoinfection. From the epidemiological point of view, the most important pathogenic species is *Clostridium botulinum.* This pathogen’s occurrence was reported in many publications as a potential risk factor for infant botulism. According to Aureli et al., 59.2% [6] of cases of infant botulism in Europe were associated with honey consumption. On the basis of epidemiological reports, the World Health Organization has recommended that infants under one year of age should not be given honey [7,8]. This variant of botulism has the form of a toxicoinfection, which differs clinically from foodborne botulism. The immaturity of the child’s intestinal microflora enables *C. botulinum* spores to germinate and produce BoNTs in situ after a baby consumes contaminated honey. The course of disease can be slow with prolonged constipation or can lead to sudden death [1]. The first infant botulism case to be recognized as a new form of botulism occurred in 1976 in the USA [9,10], and from that time infant botulism cases have been reported in 26 countries representing four continents. The largest number of cases were noted in the USA, Argentina, Italy, Canada, and Japan. In addition to BoNT produced by *C. botulinum*, toxins released by some unusual *Clostridium baratii* and *Clostridium butyricum* strains were also reported as causative agents of infant botulism [11]. Infant botulism cases mainly present during the first 6 months of life [12]. The incubation period of infant botulism is reported to be a minimum of 3 days. Usually, the first symptom of illness is constipation, which can last for 3 or more days, and peristalsis inhibition is also observed. Subsequently, a slow course is observed as is general muscular weakness instead of paralysis. The culmination of paresis usually occurs in one or two weeks. Most frequently, affected infants show normal reactions to environmental stimuli [11].

After *C. botulinum*, *Clostridium perfringens* strains are considered to be the second most common bacterial cause of foodborne illness in the USA, causing one million cases each year. The occurrence of this species and potential risk associated with prevalence of this microorganism in honey is rarely described in literature [13]. *C. perfringens* is an anaerobic, Gram-positive, spore-forming bacillus [14] frequently isolated from soil and is a natural inhabitant of human and other warm-blooded mammalian microflora [15]. The strains responsible for causing disease in humans have a chromosomally encoded *cpe* gene [14]. *C. perfringens* is characterized by rapid growth in food and vegetative cells can double in number even in 10 min [16]. Its growth is characterized by intensive production of toxic gases (e.g., sulfite). Therefore, this opportunistic pathogen could cause various histotoxic infections in humans, such as gas gangrene in contaminated wounds, gastroenteritis (including foodborne and non-foodborne diarrhea) in human adults, necrotic enteritis (NE) in animals, and recently reported NEC (necrotizing enterocolitis) in pre-term infants [13]. In general, the occurrence of this microorganism is an indicator for hygienic aspects of food product processing distribution of and environmental contamination with clostridia, and fecal distribution of spores [17]. *Clostridium perfringens* is ubiquitous in the environment, and can be a normal component of intestinal microbiota. When the intestinal microbiome balance is disturbed by sudden change of diet, antibiotic therapy, or infection by parasites, *C. perfringens* can cause enteritis in a wide range of animals [18], as well as human beings [19]. This pathogen can produce a wide range of extracellular toxins and hydrolytic enzymes (>20), is able to survive in aerobic environments (i.e., oxygen tolerance). *C. perfringens* strains are differentiated on basis of the ability to produce toxins: α-toxin, β-toxin, ε-toxin and ι-toxin, enterotoxin (CPE) and NetB. Recently, the number of toxinotypes was updated from five (A–E) to seven (A–G) [13].

The aim of our study was to assess the occurrence of BoNT-producing clostridia and *C. perfringens* strains in honey samples from Kazakhstan.

## 2. Results

### 2.1. Culture Process

#### 2.1.1. In-House Validation of Culture Enrichment Method

Recovery of *C. botulinum* NCTC 887 (toxin type A) spores was possible at each examined level of contamination (10^0^, 10^1^ and 10^2^ spores/g) and from all the samples of contaminated honey. The characteristic real-time PCR and PCR products for *ntnh* and *bont/A* genes occurrence were noticed after culturing in TPGY (Tryptone Peptone Glucose Yeast Extract) broth and after differentiation on Willis–Hobbs and FAA (Fastidious Anaerobe Agar) agar media. Characteristic metabolic features of recovered reference strain were observed as a proteolytic and lipolytic activity. The Gram staining results indicated the occurrence of bacilli with tendency to produce subterminally located spores.

#### 2.1.2. Culture Identification

Among the 197 samples examined, growth of isolates was observed in five of the samples which was a sufficient characteristic to classify them as microorganisms suspected to be BoNT-producing clostridia (K1–5). The five cultured isolates showed the ability to produce subterminal spores, from which proteolytic and lipolytic activity was observed in three of isolates.

The strains, with characteristic phenotypic features for *C. perfringens* species, were isolated from 18 samples. The cultured isolates created regular, round colonies surrounded by precipitation zones indicating on lecitinolytic properties.

**Figure 1:**
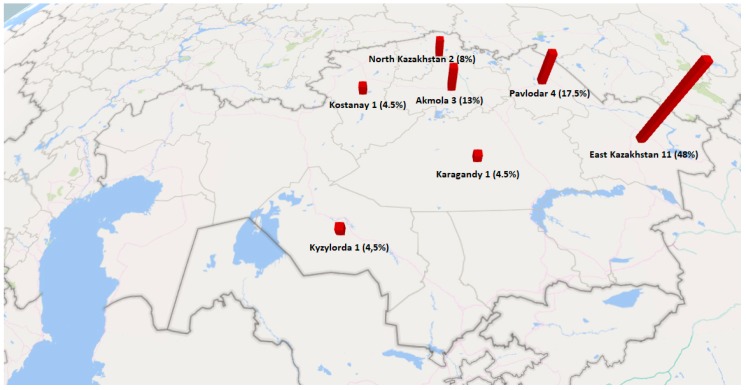
Prevalence of clostridia in honey samples collected from individual Kazakh provinces. The highest number of isolates was recovered from East Kazakhstan.

**Figure 2:**
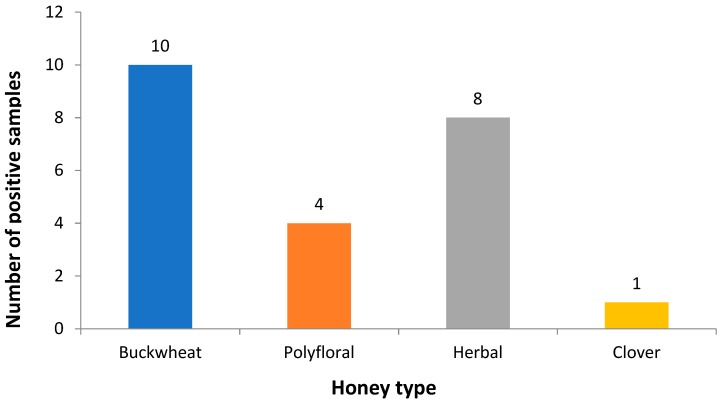
Prevalence of clostridia by honey type. The highest number of clostridia isolates was detected in buckwheat honey samples.

The highest number of clostridia strains were isolated from samples collected in the East Kazakhstan province, where prevalence was 11/23 (48%) (Table 1, Figure 1). Clostridia were isolated from buckwheat honey in 10/23 samples (43.5%), herbal honey in 8/23 (35%), polyfloral honey in 4/23 (17%) and clover honey in 1/23 (4.5%) (Table 1, Figure 2).

### 2.2. Clostridium Botulinum Detection

#### 2.2.1. Real-Time PCR Analysis Results

Positive results of real-time PCR analysis were obtained only for reference *C. botulinum* strains (NCTC 887, NCTC 3815, NCTC 8266, and NCTC 10281) and for one examined sample (0.5%). As mentioned above, only 1 out of 5 of suspected strains carried the *ntnh* gene (Table 2). *C. botulinum* was isolated from buckwheat honey collected in the area of East Kazakhstan province (Table 1).

#### 2.2.2. mPCR for Bont Genes Detection

Gene *bont/A* was detected in an isolate from one sample (Table 2). This strain, showing proteolytic properties, was classified to toxin type A group I.

### 2.3. Clostridium Perfringens Detection

*C. perfringens* were isolated from 18/197 (9%) samples and multiplex PCR (mPCR) analysis indicated on the *cpa* gene occurrence in each examined strain and led to their classification as toxin type A with the ability to produce α toxin (Table 3). None of examined isolates possessed the *cpe* genes determining enterotoxin production. Among isolated strains of *C. perfringens*, 9/18 (50%) were isolated from the samples collected in East Kazakhstan and most originated from buckwheat honey (Table 1).

### 2.4. Detection of Other Spore-Forming Anaerobes by Sequence Analysis of 16S rDNA Amplicons

Sequencing using the BLAST (Basic Local Alignment Search Tool) algorithm showed occurrence of other anaerobe species. The similarity between analyzed sequences of examined anaerobes and those deposited in GenBank ranged from 94 to 99%. Sequence analysis showed the occurrence of the species described in Table 2, including the *C. botulinum*-related *C. sporogenes* and *C. beijerinckii* (K2–K5, Table 4).

## 3. Discussion

The level of contamination of Kazakh honey by *C. botulinum* was relatively low, with its detection in only one sample (0.5%). This result correlated with a previous study on the prevalence of *C. botulinum* spores in Kazakh honey evaluating it at 0.9% (1/120) [20]. The results mentioned seem to be lower than those obtained from the countries of central and western Europe. Nevas et al. [1] reported contamination of Swedish honey samples at the level of 2% (1/50). Midura et al. [8] noticed 10% prevalence of spores in samples collected in the USA. Nakano et al. [21] described the occurrence of *C. botulinum* spores in Japanese honey samples at the level of 8.5%, whereas in another study by him and co-authors [21] positive samples were estimated at the level of 31% (11/36). Küplülü et al. [22] reported a 12.5% level in Turkish samples after examination for *C. botulinum* spores. Samples of honey from Poland were examined by two research groups; the occurrence of contamination was estimated at 2% by Grenda et al. [23], while the level described by Wojtacka et al. [24] was significantly higher and reached 22%. Lithuanian honey sold directly by the apiarist was also examined by Wojtacka et al. [25] and the level of 60% positive samples is the highest reported in literature until now. The natural prevalence of *C. botulinum* spores in food is reported to be between 10 and 1000 spores/kg [26]. The highest number of anaerobic isolates were detected in the samples of buckwheat honey, at 10/26 (38%). This type of honey was reported by Różańska (who conducted analysis of the microbiological quality of Polish honey) to be one of the most highly contaminated by anaerobes at 32.5% of examined samples positive [27].

The dilution centrifugation (DC) honey sample preparation method described in our study has been referenced several times in literature [28,29,30,31], and was recommended by Küplülü et al. [22]. The authors found this method more efficient than using direct addition (DA) or supernatant filtration (SF). According to Küplülü et al. [22], the DA and SF methods can produce false negative results more frequently. The set of molecular methods used in this study could be applied for specific detection of *C. botulinum* in various matrices. According to Grenda et al. [32], the limit of detection of the multiplex PCR used in our study expressed as LOD_50_ for food samples was estimated as follows: 0.034 (0.021–0.056) spore/g for toxin type A, 0.035 (0.022–0.054) spore/g for toxin type B, 0.094 (0.069–0.129) spore/g for toxin type E, and 0.102 (0.062–0.168) spore/g for toxin type F. Besides being included in this study, the results of validation for direct detection of the *ntnh* gene using real-time PCR were previously described by Grenda and Kwiatek [33]. The detection limit of the direct real-time PCR for contaminated food samples was estimated at 232 fg of DNA. The same real-time PCR was described by Raphael and Andreadis in 2007 [34], who reported that using purified DNA, the assay had a sensitivity of 4–95 genome equivalents. *C. botulinum* type A was detected directly in spiked stool samples at 10^2^–10^3^ CFU/mL.

Laboratory detection of *C. botulinum* spores is a difficult challenge because of the heterogenicity of this microorganism. Moreover, some strains of Clostridia exist which are biochemically similar to *C. botulinum* but not able to produce botulinum toxins. High heterogeneity is the reason for *C. botulinum* strains’ classification to four metabolic groups. Other microorganisms which are considered as non-toxinogenic are also related to these groups (excluding some toxinogenic strains of *C. butyricum*, *C. baratii* and the recently described *C. sporogenes*). According to their metabolic properties and 16S rRNA analysis, *C. sporogenes* is considered to be related to group I, *C. beijerinckii* and *C. butyricum* to group II, *C. novyi* to group III, and *C. subterminale* and *C. schirmacherense* to group IV [35,36,37]. In the honey samples, we detected some strains related to *C. botulinum* which did not possess the *ntnh* and *bont* genes. These strains were classified to *C. beijerinckii* and *C. sporogenes* species according to the results of the 16S rDNA analysis. Research conducted on the group I, II and III genomes showed that *bont* genes are frequenly plasmid-borne. Group III contains numerous plasmids carrying different toxin genes. These genes could be also found in other clostridia and some are able to move among different plasmids though the same physiological group. Horizontal transfer could be observed within and between species of *Clostridium*. Mobile element occurrence, particularly group III genomes, is linked with plasticity of genome and gene transfer events mobility [38].

Besides *C. botulinum*, unusual strains from related species have been described which were able to produce BoNTs and were causative agents of human botulism cases. These include the strains of *C. baratii*, *C. butyricum*, *C. argentinense*, and *C. sporogenes*. Botulinum cluster genes were also detected in Gram–negative bacteria. Evidence was cited taking the example of *Chryseobacterium piperi* isolated from freshwater sediments, however biological activity of *bont*-related genes was not noted [39]. Zhang et al. [40] described BoNT-related toxin (BoNT/En) produced by *Enterococcus faecium*. The obtained results indicated 29–38% identity with other BoNTs. However, the new toxin described was not toxic to mice [41].

The level of honey contamination with *C. botulinum* spores may depend on the harvesting region and the care invested in hygiene at harvest. BoNT–producing clostridia commonly occur in soil, sediments, and in food, however usually in the form of very resistant spores [37]. There is a recognizable geographical variation in the prevalence of A and B toxin types. Type A occurrence is predominant in the western part of the USA, and soil in this area is close to alkaline (the average pH is 7.5) and organic content is low. *C. botulinum* type B is more frequently isolated in the eastern part of the USA and Central and Western Europe. Type B is mostly isolated from more acidic soil and sediments with average pH 6.5. In such soil, the organic matter content is higher, which is characteristic for pastures and fields [42]. The average pH value of Kazakh soil is alkaline (the average pH is about 8.0) and the organic matter level is low [43]. The dry continental climate is an unfavorable condition for crops cultivation, especially in the south of the country, where deserts and semi deserts feature. Further to the north–east, flora become much more variable [44]. Climate conditions could correlate with the level of *C. botulinum* prevalence. The incidence of infant botulism could be associated with the density of spores in a given area and their geographical distribution; cases may occur after soil disruption by farming and possible transfer to pollen, and subsequently to honey. Strength of winds could have an influence on spore transfer, and violent natural phenomena could trigger more frequent outbreaks, e.g., as was the case with infant botulism cases after the Northridge earthquake in 1994 [11]. A dry climate with high winds is typical for Kazakhstan [44]. Infant botulism cases could potentially appear in the area of East Kazakhstan where agriculture activity is most dynamic and distribution of clostridia spores seems to be the densest. However, evidence of cases has not been reported up to the time of this study. According to Nevas et al. [1], secondary factors which also have significant influence on the presence of *C. botulinum* spores in honey are the size of the extractor.

The results of our study indicated a high prevalence of *C. perfringens* spores in Kazakh honey samples. It was noted that *C. perfringens* type A was isolated from 9% of examined samples. Generally, there are only a few publications concerning the occurrence of this microorganism in honey. Tomassetti et al. [4] provided information about *C. perfringens* in jar and comb honey samples.

The authors estimated occurrence of this relative pathogen at 16.2% in jar honey and at 11.3% in comb honey samples. Grenda et al. reported that the *C. perfringens* occurrence was noted in 27.5% (66/240) samples of Polish honey, collected after an extraction process [45]. A recent study by Kiu et al. [46] showed *C. perfringens* strains revealed a high diversity with only 12.6% of genes occurring common to each genome. In this study there appeared the suggestion that diverse genetic variations could be determined by HGT (horizontal gene transfer), mainly prophage insertion within the Clustered Regularly Interspaced Short Palindromic Repeat-free (CRISPR-free) genomes (no single CRISPR prophage defence system was detected with more than one occurrence for a 70% proportion of genomes). These results give new insight into the epidemiology of *C. perfringens*. Further exploration of the genetic diversity of Kazakh honey isolates and comparison with strains isolated from other regions (e.g., western countries) should be undertaken with the emphasis on analysis of the occurrence of genes determining pathogenicity and antibiotic resistance. Epidemiological status of diseases caused by *C. perfringens* in Kazakhstan is unknown and data about *C. perfringens* prevalence in the environmental samples are limited. Published reports indicate the prevalence of this microorganism in wild animals. One of these reports indicates *C. perfringens* type A (able to produce α toxin), as a potential causative factor of Saiga Antelopes high mortality observed in the years 2010–2013 in West Kazakhstan and Kostanai regions [47]. *C. perfringens* was isolated from internal organs and the blood of dead animals. Observed mortality was linked by authors with causative activity of *C. perfringens* type A. However, isolates were able to produce only α toxin and any other virulence factors had not detected [47]. Another outbreak, concerning *C. perfringens* as a causative agent of toxemia in wild boar was reported in Zhaosu County, Xinjiang Province, PRC, near the border with Kazakhstan (East Kazakhstan region) [48]. *C. perfringens* type A and type C was successfully isolated from the collected intestine samples. Authors indicated that wild boars could be a natural vector of this microorganism [48]. Our study indicated the most frequent prevalence of *C. perfringens* isolated from honey in the East Kazakhstan. In analyzed honey samples, only type A strains were detected with ability to produce α toxin. Food-poisoning is associated generally with occurrence of enterotoxic strains able to produce CPE characteristic for toxotype F strains (previously named as CPE-positive type A) although CPE can also be produced by certain type C, D and E strains. Distribution of *C. perfringens* in environmental samples collected in Kazakhstan is unknown. Potential factors favoring the occurrence of this microorganism could be connected with the distribution of wild animals and organic matter in a given area. These opportunistic pathogens could start the disease progress in oxygenic environments (i.e., adult/pre-term infant intestines), and tissues exposed to oxygen activity (such as gas gangrene), and this could trigger bacterial host-to-host transmission. According to Matches et al., *C. perfringens* is also abundant in human feces and domestic waste contains large numbers of this microorganism [49]. East and Central Kazakhstan seems to be the most industrial and populated area with the highest number of apiaries [44]. The prevalence in honey of this microorganism seems to be the most probable on the mentioned regions.

The *C. botulinum* prevalence in honey samples from Kazakhstan (0.5%) is lower than that of samples collected from other regions (2–60%) [1,8,21,22,23,24,29]. According to the literature, the conditions of honey harvest, extraction, type of soil, climate, intensiveness of agricultural production, and occurrence of environmental phenomena have influence on clostridia spore content in honey samples. In the case of Kazakh honey, which factors bear upon spore occurrence have not been determined. In order to know the routes of *Clostridium* sp. transmission to honey samples, a risk analysis should be conducted. Kazakhstan has not been investigated for the occurrence and epidemiology of this genus. Our study is the first survey on BoNT-producing clostridia and *C. perfringens* prevalence in Kazakh honey samples.

## 4. Materials and Methods

### 4.1. Honey Samples

Honey is produced almost everywhere in the Kazakh Republic, however, the biggest producer is the East Kazakhstan region (70%) [44]. In order to increase the profitability of beekeeping, many apiary owners have abandoned their stationary hive placement and moved to a nomadic pattern. As in the southern regions all agricultural and horticultural crops bloom a few weeks earlier than those in the northern ones, the transport of beehives from one region to another extends the time of honey harvesting from plants of the same species.

**Figure 3:**
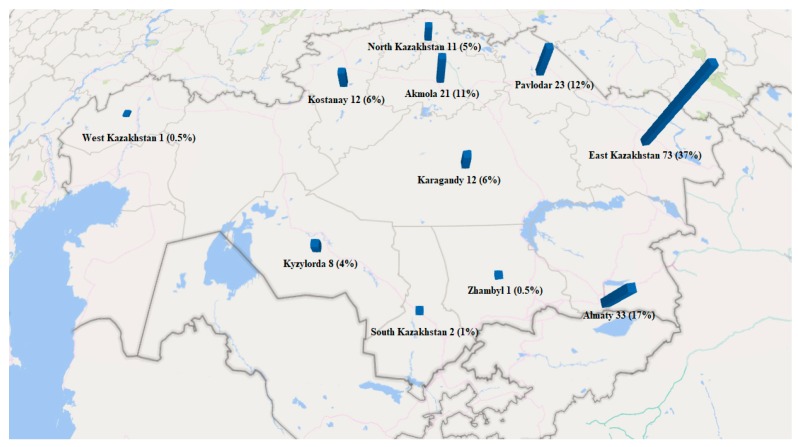
Number of samples collected from particular provinces of Kazakhstan. The highest number of samples were collected from the East Kazakhstan region where highest intensity agriculture is practiced.

**Figure 4:**
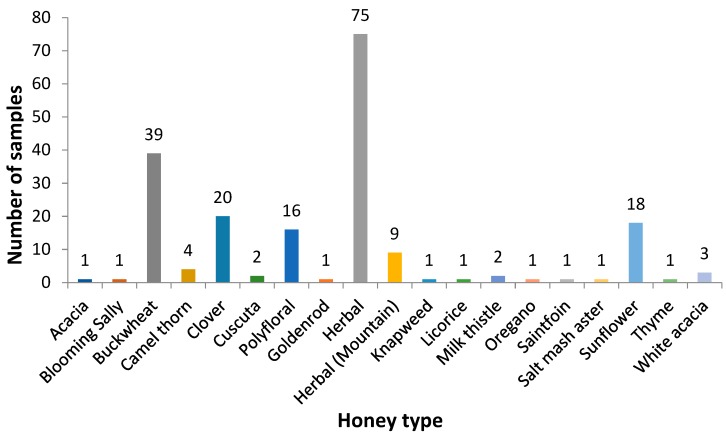
Sample numbers of particular honey types. The best represented of them were herbal (75, 38%), buckwheat (39, 20%), clover (20, 10%), sunflower and polyfloral (8%).

Studies were conducted using 197 samples of honey from Kazakhstan. Honey samples were collected from both field and roadside apiaries, honey fairs, and markets in different regions of Kazakhstan (Table 5, Figure 3), and variety in the types of honey collected was designed in (Table 3, Figure 4. Sampling of honey was carried out in the summer and autumn periods. Most of the samples were collected from the region of East Kazakhstan (n = 73) (37%) (Table 5, Figure 3). The honey varieties amassed in the greatest numbers were herbal 75 (38%), buckwheat 39 (20%), clover 20 (10%), sunflower and polyfloral 16 (8%) (Table 5, Figure 4).

Analysis was conducted using culturing, molecular biology methods (PCR and real-time PCR) and sequence analysis.

### 4.2. Enrichment Cultures

Each 10 g honey sample was diluted in 90 mL of distilled water with addition of 1% Tween 80 and mixed until homogeneity was achieved. The obtained solution was centrifuged at 9000× *g* for 30 min (Eppendorf Minispin, Sigma, Hamburg, Germany). Subsequently, the pellet obtained was inoculated into tubes with 90 mL TPGY broth (50 g/L casein enzymic hydrolysate, 5 g/L peptic digest of animal tissue, 20 g/L yeast extract, 4 g/L dextrose, and 1 g/L sodium thioglycolate with a final pH of 7.0 ± 0.2 at 25 °C). After inocula preparation, the thermal treatment was conducted at 70 °C for 15 min. Inoculated samples were subjected to incubation at 30 °C for seven days [21]. After this period the growth of anaerobes was assessed and 1 mL of liquid culture from 90 mL tubes was inoculated to the tubes with 10 mL fresh TPGY broth for an additional two days of incubation. Subsequently 2–3 drops of these cultures were spread on Willis–Hobbs agar (10 g/L peptic digest of animal tissue, 10 g/L meat extract, 5 g/L sodium chloride, 12 g/L lactose, 0.032 g/L neutral red, 10 g/L skim milk powder, 2 g egg yolk powder and 10 g/L agar with a final pH of 7.0 ± 0.2 at 25 °C) and FAA (23 g/L peptone, 5 g/L sodium chloride, 1 g/L soluble starch, 0.4 g/L sodium bicarbonate, 1 g/L glucose, 1 g/L sodium pyruvate, 0.5 g/L L-cysteine HCl × H_2_O, 0.25 g/L sodium pyrophosphate, 1 g/L L-arginine, 0.5 g/L sodium succinate, 0.01 g/L hemin, 0.001 g/L vitamin K, 2 g egg yolk powder, and 12 g/L agar with a final pH of 7.2 ± 0.2 at 25 °C) and incubated anaerobically at 30 °C for 48 h. The grown colonies were evaluated for their surface, shape, size, color and lipolytic, proteolytic or lecitinolytic features, and isolates were additionally subjected to Gram staining. All cultures were prepared in duplicate.

#### In-House Validation of Culture Enrichment Process 

The culture procedure was validated by using polyfloral honey samples contaminated by *C. botulinum* NCTC 887 spores obtained according to method described by Fletcher et al. in 2008 [50]. Contamination was introduced at 10^−1^, 10^0^ spores/g and 10^1^ spores/g. Validation was carried out using five samples per analyzed level.

### 4.3. Nucleic Acids Isolation

#### DNA Isolation

DNA was isolated from 1 mL of liquid cultures and from several colonies obtained from agar plates. The DNA was extracted with a Genomic Mini AX Bacteria kit (A&A Biotechnology, Gdynia, Poland) according to the manufacturer’s instructions. The amount of DNA used in the PCR reaction varied between 1 and 25 ng. The extracted DNA was frozen at −20 °C or directly subjected to PCR analysis.

### 4.4. Clostridium Botulinum Identification

Reference strains of different clostridia species were used as positive controls: *C. botulinum* NCTC 887 (type A), *C. botulinum* NCTC 3815 (type B), *C. botulinum* NCTC 8266 (type E), and *C. botulinum* NCTC 10281 (type F).

#### 4.4.1. ntnh Gene Detection by Real-Time PCR

The set of 7 primers and TaqMan probe were used for detection of the *ntnh* gene according to Raphael and Andreadis 2007 [34]. The reaction was conducted with subsequent concentrations of reagents: 5 μL of DNA, 4 μL of LightCycler TaqMan Master (Roche, Basel, Switzerland), 0.7 μM of each primer and 0.24 μM of NTNH410 TaqMan probe. The real-time PCR was performed using a LightCycler 2.0 thermocycler (Roche, Basel, Switzerland) on the following thermal cycling profile: 10 min at 95 °C as initial denaturation and 40 cycles of denaturation at 95 °C for 15 s, annealing at 42 °C for 15 s, and elongation at 55 °C for 1 min. Validation results of this method for food samples are described in a previous publication [33].

#### 4.4.2. Bont/A, Bont/B, Bont/E, and Bont/F GENES detection

A, B, E and F toxin types were determined in accordance with the method of De Medici et al., 2009 [51]. The reaction volume equaled 25 µL and comprised 5 μL of DNA, 2.5 μL of 10× Taq buffer with KCl (Thermo Fisher Scientific, Waltham, MA, USA), 4 mM of MgCl_2_, 200 μM of dNTP, 0.3 µM of each primer and Taq polymerase 1.25 U/25 μL (Thermo Fisher Scientific, Waltham, MA, USA). The validation results of this method for food samples were described in our previous publication [32]. Products were detected on agarose gel.

### 4.5. Clostridium Perfringens Detection

*C. perfringens* ATCC 13124 strain was used as the positive control in the experiments. Isolates from Willis–Hobbs or FAA agars suspected to be *C. perfringens* were subjected to DNA preparation and detection with the mPCR method according to Baums et al., 2004 [52] with Kukier and Kwiatek’s modification [53]. This method enabled simultaneous α, β, ε, ι, β2 and CPE gene detection and subsequent determination of the toxin types of themicroorganism. Detection was performed on agarose gel.

### 4.6. Detection of Other Spore-Forming Anaerobes by Amplification and Sequencing of 16S rDNA

For the characterization of 16S rDNA in unidentified anaerobic strains (grown on FAA and Willis–Hobbs agar), the PCR method with primers according to Vaneechoutte et al. [54] was used. The reaction volume was 25 µL and the reagent constituents were 5 µL of DNA matrix, 2.5 μL 10× Taq buffer with KCl (Thermo Fisher Scientific, Waltham, MA, USA), 4 mM MgCl_2_, 200 μM dNTP, 0.3 µM of each primer and 1.25 U/25 μL Taq polymerase. The reaction was staged as follows: after initial denaturation at 95 °C for 5 min, there were 35 cycles of denaturation at 95 °C for 45 s, annealing at 55 °C for 45 s, and extension at 72 °C for 1 min. Finally, a 10-min extension period at 72 °C was included. The obtained length of products was about 1500 bp. Sequence analysis of the PCR products was commissioned from Genomed (Warsaw, Poland). The FASTA (text-based format for representing either nucleotide sequences or amino acid sequence) files produced thereby were analyzed with the BLAST (NCBI—National Center for Biotechnology Information, Bethesda, Maryland, USA) algorithm against the nucleotide collection database. A BLAST search was performed for comparison of amplicons with the highest sequence similarity scores from NCBI.

### 4.7. Electrophoresis of PCR Products

Agarose gels were prepared at a concentration of 2% in 1× Tris-acetate-EDTA (TAE) and stained with SimplySafe (EURx, Gdańsk, Poland) nucleic acid stain. Electrophoresis was carried out in 1× TAE buffer for 1.5 h at 100 V. The molecular weight of obtained products was evaluated using GeneRulerTM 100 bp DNA Ladder Plus (Thermo Fisher Scientific, Waltham, MA, USA).

## Figures and Tables

**Table 1:** Clostridia isolates from Kazakh honey samples.

Province	Number of *C. botulinum* Isolates (Honey Type)	Number of Isolates Related to *C. botulinum* Species (Honey Type)	Number of *C. perfringens* Isolates (Honey Type)	Total in Provinces (%)
East Kazakhstan	1 (Buckwheat)	1 (Herbal)	4 (Buckwheat), 2 (Polyfloral), 3 (Herbal)	11 (48%)
Almaty	-	-	-	-
Pavlodar	-	-	2 (Buckwheat), 1 (Clover), 1 (Polyfloral)	4 (17.5%)
Akmola	-	2 (Buckwheat)	1 (Herbal)	3 (13%)
Kostanay	-	1 (Buckwheat)	-	1 (4.5%)
Karagandy	-	-	1 (Herbal)	1 (4.5%)
North Kazakhstan	-	-	1 (Polyfloral), 1 (Herbal)	2 (8.5%)
Kyzylorda	-	-	1 (Herbal)	1 (4.5%)
South Kazakhstan	-	-	-	-
Zhambyl	-	-	-	-
West Kazakhstan	-	-	-	-
Total	1 (4.5%)	4 (17.5%)	18 (78%)	23 (100%)

**Table 2:** Occurrence *ntnh* and *bont* genes in isolates suspected to be BoNT—producing clostridia.

Province	Number of Suspected Isolates	Number of Isolates Carrying Particular Genes				
		*ntnh*	*bont*/*A*	*bont*/*B*	*bont*/*E*	*bont*/*F*
East Kazakhstan	2	1	1	-	-	-
Akmola	2	-	-	-	-	-
Kostanay	1	-	-	-	-	-
Total	5	1	1	-	-	-

**Table 3:** Occurrence of *cpa*, *cpb*, *etx*, *iap*, *cpe* and *cpb2* genes in isolates suspected of belongings to *C. perfringens* species.

Province	Number of Suspected Isolates	Number of Isolates Carrying Particular Genes					
		*cpa*	*cpb*	*etx*	*iap*	*cpe*	*cpb2*
East Kazakhstan	9	9	-	-	-	-	-
Pavlodar	4	4	-	-	-	-	-
Akmola	1	1	-	-	-	-	-
Karagandy	1	1	-	-	-	-	-
North Kazakhstan	2	2	-	-	-	-	-
Kyzylorda	1	1	-	-	-	-	-
Total	18	18	-	-	-	-	-

**Table 4:** Sequencing results of 16S rDNA PCR amplicons.

Isolate	Similar Sequence from GenBank (NCBI)	Sequence ID	% Similarity	Origin
K2	*Clostridium sporogenes* strain A4 16S rRNA	KY910419.1	98%	Akmola province/buckwheat honey
K3	*Clostridium sporogenes* strain 1101M 16S rRNA	KU950270.1	99%	Akmola province/buckwheat honey
K4	*Clostridium beijerinckii* strain JCM 7837 16S rRNA	LC258130.1	95%	East Kazakhstan province/herbal honey
K5	*Clostridium beijerinckii* strain JCM 7837 16S rRNA	LC258130.1	94%	Kostanay province/buckwheat honey

**Table 5:** Honey samples collected from various provinces of Kazakhstan.

Province	Number of Samples	Honey Type
East Kazakhstan	73 (37%)	Acacia (1), Buckwheat (19), Clover (5), Polyfloral (4), Herbal (31), Herbal (Mountain) (2), Milk thistle (1), Oregano (1), Sunflower (7), White acacia (2)
Almaty	33 (17%)	Buckwheat (2), Camel thorn (2), Clover (3), Polyfloral (1), Herbal (14), Herbal (Mountain) (7), Salt marsh aster (1), Sunflower (3)
Pavlodar	23 (12%)	Buckwheat (7), Clover (5), Polyfloral (2), Herbal (5), Sunflower (4)
Akmola	21 (11%)	Buckwheat (4), Clover (3), Polyfloral (2), Herbal (7), Sainfoin (1), Sunflower (2), White acacia (1), Camel thorn (1)
Kostanay	12 (6%)	Buckwheat (2), Clover (1), Polyfloral (2), Herbal (6), Sunflower (1)
Karagandy	12 (6%)	Buckwheat (3), Clover (1), Polyfloral (2), Goldenrod (1), Herbal (3), Milk thistle (1), Thyme (1)
North Kazakhstan	11 (5%)	Blooming Sally (1), Buckwheat (2), Clover (1), Polyfloral (1), Herbal (5), Sunflower (1)
Kyzylorda	8 (4%)	Camel thorn (1), Cuscuta (2), Polyfloral (1), Herbal (3), Licorice (1)
South Kazakhstan	2 (1%)	Clover (1), Polyfloral (1)
Zhambyl	1 (0.5%)	Knapweed (1)
West Kazakhstan	1 (0.5%)	Herbal (1)
Total	197 (100%)	

## References

[1] Nevas M., Lindström M., Hautamäki K., Puoskari S., Korkeala H. (2005). Prevalence and diversity of *Clostridium botulinum* types A, B, E and F in honey produced in the Nordic countries. Int. J. Food Microbiol..

[2] Olaitan P.B., Adeleke O.E., Ola I.O. (2007). Honey: A reservoir for microorganisms and an inhibitory agent for microbes. Afr. Health Sci..

[3] Sereia M.J., Alves E.M., de Toledo V.A.A., Marchini L.C., Faquinello P., Sekine E.S., Wielewski P. (2017). Microbial flora in organic honey samples of Africanized honey bees from Parana River islands. Food Sci. Tech.-Braz..

[4] Tomassetti F., Milito M., Dell’Aira E., de Santis L., Migliore G., Formato G. (2009). Microbiological comparison between honey in jar and honey in comb for human. Ital. J. Food Saf..

[5] Wen Y., Wang L., Jin Y., Zhang J., Su L., Zhang X., Zhou J., Li Y. (2017). The microbial community dynamics during the vitex honey ripening process in the honeycomb. Front. Microbiol..

[6] Aureli P., Franciosa G., Fenicia L. (2002). Infant botulism and honey in Europe: A commentary. Pediatr. Infect. Dis. J..

[7] Abdulla C.O., Ayubi A., Zulfiquer F., Santhanam G., Ahmed M.A.S., Deeb J. (2012). Infant botulism following honey ingestion. BMJ Case Rep..

[8] Midura T.F. (1996). Update: Infant Botulism. Clin. Microbiol. Rev..

[9] Midura T.F., Arnon S.S. (1976). Infant botulism. Identification of *Clostridium botulinum* and its toxins in faeces. Lancet.

[10] Pickett J., Berg B., Chaplin E., Brunstetter-Shafer M.A. (1976). Syndrome of botulism in infancy: Clinical and electrophysiologic study. N. Engl. J. Med..

[11] Fenicia L., Anniballi F. (2009). Infant botulism. Ann. Ist. Super. Sanità.

[12] Weissenstein A., Villalon G., Luchter E., Bittmann S. (2018). Medical honey and the role in pediatric emergency wound management. J. Pediatr. Dis..

[13] Kiu R., Hall L.J. (2018). An update on the human and animal enteric pathogen *Clostridium perfringens*. Emerg. Microbes Infect..

[14] McClane B.A., Doyle M.P., Beuchat L.R. (2007). Clostridium perfringens. Food Microbiology: Fundamentals and Frontiers.

[15] Brynestad S., Granum P.E. (2002). Clostridium perfringens and foodborne infections. Int. J. Food Microbiol..

[16] Labbe R.G., Juneja V.K., Riemann H.P., Cliver D.O. (2006). *Clostridium perfringens* gastroenteritis. Foodborne Infections and Intoxications.

[17] Grass J.E., Gould L.H., Mahon B.E. (2013). Epidemiology of foodborne disease outbreaks caused by *Clostridium perfringens*, United States, 1998–2010. Foodborne Pathog. Dis..

[18] Uzal F.A., Saputo J., Sayeed S., Vidal J.E., Fisher D.J., Poon R., Adams V., Fernandez-Miyakawa M.E., Rood J.I., McClane B.A. (2009). Development and application of new mouse models to study the pathogenesis of Clostridium perfringens type C Enterotoxemias. Infect. Immun..

[19] Qiu H., Chen F., Leng X., Fei R., Wang L. (2014). Toxinotyping of Clostridium perfringens fecal isolates of reintroduced P`ere David’s deer (Elaphurus davidianus) in China. J. Wildl. Dis..

[20] Mustafina R., Maikanov B., Wiśniewski J., Tracz M., Anusz K., Grenda T., Kukier E., Goldsztejn M., Kwiatek K. (2015). Contamination of honey produced in the Republic of Kazakhstan with *Clostridium botulinum*. Bull. Vet. Inst. Pulawy.

[21] Nakano H., Sakaguchi G. (1991). An unusually heavy contamination of honey products by *Clostridium botulinum* type F and *Bacillus alvei*. FEMS Microbiol. Lett..

[22] Küplülü Ö., Göncüoğlü M., Özdemir H., Koluman A. (2006). Incidence of Clostridium botulinum spores in honey in Turkey. Food Control.

[23] Grenda T., Grabczak M., Sieradzki Z., Kwiatek K., Pohorecka K., Skubida M., Bober A. (2018). *Clostridium botulinum* spores in Polish honey samples. J. Vet. Sci..

[24] Wojtacka J., Wysok B., Lipiński Z., Gomółka-Pawlicka M., Rybak-Chmielewska H., Wiszniewska-Łaszczych A. (2016). *Clostridium botulinum* spores found in honey from small apiaries in Poland. J. Apic. Sci..

[25] Wojtacka J., Wysok B., Kabašinskienė A., Wiszniewska-Łaszczych A., Gomółka-Pawlicka M., Szteyn J., Malakauskas M., Migowska-Calik A. (2017). Prevalence of *Clostridium botulinum* Type A, B, E and F Isolated from Directly Sold Honey in Lithuania. J. Agric. Sci. Technol..

[26] Lindström M., Korkeala H. (2006). Laboratory diagnostics of botulism. Clin. Microbiol. Rev..

[27] Różańska H. (2011). Microbial quality of Polish honey. Bull. Vet. Inst. Pulawy.

[28] Midura T.F., Snowden S., Wood R.M., Arnon S.S. (1979). Isolation of *Clostridium botulinum* from honey. J. Clin. Microbiol..

[29] Nakano H., Okabe T., Hashimoto M., Sakaguchi G. (1990). Incidence of *Clostridium botulinum* in honey of various origins. Jpn. J. Med Sci. Biol..

[30] Huhtanen C.N., Knox D., Shimanuki H. (1981). Incidence and origin of *Clostridium botulinum* spores in honey. J. Food Prot..

[31] Schocken-Iturino R.P., Carneiro M.C., Kato E., Sorbara J.O.B., Rossi O.D., Gerbasi L.E.R. (1999). Study of the presence of the spores of *Clostridium botulinum* in honey in Brazil. FEMS Immunol. Med. Microbiol..

[32] Grenda T., Kukier E., Sieradzki Z., Goldsztejn M., Kwiatek K. (2012). In-house validation of multiplex PCR method for detection of *Clostridium botulinum* in food and feed. Bull. Vet. Inst. Pulawy.

[33] Grenda T., Kwiatek K. (2010). In-house validation of real-time PCR method for detection of *Clostridium botulinum* in food and feed matrixes. Bull. Vet. Inst. Pulawy.

[34] Raphael B.H., Andreadis J.D. (2007). Real-time PCR detection of the nontoxic nonhemagglutinin gene as a rapid screening method for bacterial isolates harboring the botulinum neurotoxin (A–G) gene complex. J. Microbiol. Methods.

[35] Carter A.T., Peck M.W. (2015). Genomes, neurotoxins and biology of *Clostridium botulinum* Group I and Group II. Res. Microbiol..

[36] Collins M.D., East A.K. (1998). Phylogeny and taxonomy of the food-borne pathogen *Clostridium botulinum* and its neurotoxins. J. Appl. Microbiol..

[37] Scalfaro C., Auricchio B., De Medici D., Anniballi F. (2019). Foodborne botulism: An evolving public health challenge. Infect. Dis. (Lond.).

[38] Skarin H., Segerman B. (2011). Horizontal gene transfer of toxin genes in *Clostridium botulinum*. Mob. Genet. Elem..

[39] Wentz T.G., Muruvanda T., Lomonaco S., Thirunavukkarasu N., Hoffmann M., Allard M.W., Hodge D.R., Pillai S.P., Hammack T.S., Brown E.W. (2017). Closed genome sequence of *Chryseobacterium piperi* strain CTMT/ATCC BAA-1782, a Gram-negative bacterium with clostridial neurotoxin-like coding sequences. Genome Announc..

[40] Zhang S., Lebreton F., Mansfield M.J., Miyashita S.I., Zhang J., Schwartzman J.A., Tao L., Masuyer G., Martinez-Carranza M., Stenmark P. (2018). Emergence of a botulinum neurotoxin-like toxin in a commensal strain of *Enterococcus faecium*. Cell Host Microbe.

[41] Popoff M.R. (2018). Botulinum neurotoxins: Still a privilege of clostridia?. Cell Host Microbe.

[42] Poulain B., Popoff M.R. (2019). Why are botulinum neurotoxin-producing bacteria so diverse and botulinum neurotoxins so toxic?. Toxins.

[43] Li L., Chen X., van der Tol C., Luo G., Su Z. (2014). Growing season net ecosystem CO_2_ exchange of two desert ecosystems with alkaline soils in Kazakhstan. Ecol. Evol..

[44] Kazakhstan in 2009. Statistical Yearbook of Kazakhstan. Statistic Agency of Republic of Kazakhstan, Astana, 2010. http://www.stat.kz/publishing/DocLib/2010/Ewegodnik_2010.pdf.

[45] Grenda T., Grabczak M., Goldsztejn M., Kozieł N., Kwiatek K., Pohorecka K., Skubida M., Bober A. (2018). Clostridium perfringens spores in Polish honey samples. J. Vet. Res..

[46] Kiu R., Caim S., Alexander S., Pachori P., Hall L.J. (2017). Probing genomic aspects of the multi-host pathogen *Clostridium perfringens* reveals significant pangenome diversity, and a diverse array of virulence factors. Front. Microbiol..

[47] Tlenchieva T.M., Sultankulova K.T., Shoraeva K.A., Strochkov V.M., Orynbaev M.B., Zaitsev V.L., Rystaeva R.A., Sandybaev N.T., Sansyzbai A.R. (2015). Detection and characteristics of alphatoxin strain *Clostridium perfringens* genetic variability, isolated from Kazakhstan Saiga in 2010–2013. Res. J. Pharm. Biol. Chem. Sci..

[48] Li M., Zhang X., Zhu L., Wang H., Zhao N., Luo J., Wang C., Wang Y., Liu Y., Zhou W. (2017). Identification, isolation, and phylogenetic analysis of *Clostridium perfringens* type A and type C from Wild Boar (Sus scrofa) in the People’s Republic of China. J. Wildl. Dis..

[49] Matches J.R., Liston J., Curran D. (1974). *Clostridium perfringens* in the environment. Appl. Microbiol..

[50] Fletcher G.C., Youssef J.F., Lu G. (2008). Selecting Methods for Determining the Presence of BoNT Genes in New Zealand Marine Sediments.

[51] De Medici D., Anniballi F., Wyatt G.M., Lindström M., Messelhäußer U., Aldus C.F., Delibato E., Korkeala H., Peck M.W., Fenicia L. (2009). Multiplex PCR for detection of botulinum neurotoxin-producing Clostridia in clinical, food, and environmental samples. Appl. Environ. Microbiol..

[52] Baums C.G., Schotte U., Amtsberg G., Goethe R. (2004). Diagnostic multiplex PCR for toxin genotyping of *Clostridium perfringens* isolates. Vet. Microbiol..

[53] Kukier E., Kwiatek K. (2010). Occurrence of *Clostridium perfringens* in food chain. Bull. Vet. Inst. Pulawy.

[54] Vaneechoutte M., Cartwright C.P., Williams E.C., Jäger B., Tichy H.V., de Baere T., de Rouck A., Verschraegen G. (1996). Evaluation of 16S rRNA gene restriction analysis for the identification of cultured organisms of clinically important *Clostridium* species. Anaerobe.

